# Supplementary material for: The Systems Biology Research Tool: evolvable open-source software
Source: BMC Syst Biol. 2008 Jun 29;2:55. doi: 10.1186/1752-0509-2-55 (PMC2446383; doi:10.1186/1752-0509-2-55)
Supplement: Additional file 1 — SBRT Archive. An archive of the current version of the Systems Biology Research Tool. [file 1752-0509-2-55-S1.zip › sbrt-1.4.0/doc/developers_guide/api/sbrt/shell/mng/fba/IntervalVariationManager.html]

IntervalVariationManager


|  |  |  |
| --- | --- | --- |
| **Overview** \| **Package** \| **Class** \| **Use** \| **Tree** \| **Deprecated** \| **Index** \| **Help** | |  |
| **PREV CLASS**   **NEXT CLASS** | **FRAMES**    **NO FRAMES**     **All Classes** |  |
| SUMMARY: NESTED \| FIELD \| CONSTR \| METHOD | DETAIL: FIELD \| CONSTR \| METHOD |  |


---


## sbrt.shell.mng.fba Class IntervalVariationManager

```
java.lang.Object
  sbrt.shell.mng.AbstractProcessManager
      sbrt.shell.mng.fba.FbaManager
          sbrt.shell.mng.fba.IntervalVariationManager
```

**All Implemented Interfaces:**: KernelProcessManager<java.lang.String,java.lang.String>, ProcessManager<java.lang.String,java.lang.String>

---

``` public abstract class IntervalVariationManager extends FbaManager ```

This abstract class provides utitlity functions for managing flux interval
variation experiments.

**Author:**
:   This class was written and documented by Jeremiah Wright while in the
    Wagner lab.

---

| **Field Summary** | |
| --- | --- |
| `static java.lang.String` | `CV_FILE`             The keyword used to denote a constraint variation input file. |
| `static java.lang.String` | `FLUX_INTERVAL_FILE`             The keyword used to denote a flux interval input file. |

**Fields inherited from class sbrt.shell.mng.fba.FbaManager**

`CONSTRAINT_TOLERANCE, DEFAULT_CONSTRAINT_TOLERANCE, FLUX_VECTOR_FILE, FLUX_VECTOR_FILE_NAME, optionalKeywords, requiredKeywords, RXN_CATALYST_FILE, RXN_FILE, SYSTEM_SOLUTION_FILE`

**Fields inherited from class sbrt.shell.mng.AbstractProcessManager**

`ALGORITHM, COMPLETED, DATA_HEADERS, DEV_NULL, ELAPSED_TIME, FILE_FORMAT, INPUT_FILE, INPUT_FILE_FORMAT, INPUT_FILE_NAME_FILE, ITERATIONS, OUTPUT_FILE_FORMAT, OUTPUT_FILE_NAME, OUTPUT_FILE_NAME_FILE, PERCENT, PERCENT_COMPLETED, SEED, ZERO_CUTOFF`


**Constructor Summary**

`IntervalVariationManager()`


| **Method Summary** | |
| --- | --- |
| `static FluxIntervalInputFile` | `getCvInputFile(ManagerInput<java.lang.String,java.lang.String> input, java.io.PrintWriter out, Fluxome fluxome)`             Returns the constraint variation input with the name denoted by `CV_FILE` in the provided manager input. |
| `static FluxIntervalInputFile` | `getCvInputFile(java.lang.String fileName, Fluxome fluxome)`             Returns the constraint variation input file with the provided name. |
| `static FluxIntervalInputFile` | `getIntervalInputFile(java.lang.String fileName, Fluxome fluxome)`             Returns the flux interval input file with the provided name. |
| `static FluxIntervalInputFile` | `getIntervalInputFile(java.lang.String fileName, Fluxome fluxome, MapFormat<LinearComb<java.lang.String>,Interval> mapFormat)`             Returns the flux interval input file with the provided name. |
| `static FluxIntervalInputFile` | `getSvInputFile(ManagerInput<java.lang.String,java.lang.String> input, java.io.PrintWriter out, Fluxome fluxome)`             Returns the space variation input with the name denoted by `FLUX_INTERVAL_FILE` in the provided manager input. |

**Methods inherited from class sbrt.shell.mng.fba.FbaManager**

`getCatalysts, getCatalysts, getConstraintTolerance, getConstraintTolerance, getFluxome, getFluxome, getFluxome, getFluxome, getFluxome, getFluxomeSolution, getMultiFluxVectorOutputFile, getMultiFluxVectorOutputFile, getRxnNameExprFormat, getRxnNameHeaders, getRxnNameHeaders, getSingleFluxVectorOutputFile, getSingleFluxVectorOutputFile, printFluxomeInfo, writeFluxome, writeFluxome`

**Methods inherited from class sbrt.shell.mng.AbstractProcessManager**

`check, closeFile, closeRm, execute, getFileFormat, getFileFormat, getFileNameSource, getIterations, getSeed, getTextFileFormat, getTextFileFormat, getZeroCutoff, getZeroCutoff, parseInt, parseLong, printProgress, printProgress, printProgress, runProcess, setInput`

**Methods inherited from class java.lang.Object**

`clone, equals, finalize, getClass, hashCode, notify, notifyAll, toString, wait, wait, wait`

**Methods inherited from interface sbrt.shell.mng.KernelProcessManager**

`getProcess`

**Methods inherited from interface sbrt.shell.mng.ProcessManager**

`check, execute, execute, getOptionalKeys, getRequiredKeys, setInput, setInput`

**Field Detail**

### CV\_FILE

```
public static final java.lang.String CV_FILE
```

:   The keyword used to denote a constraint variation input file.

    **See Also:**: Constant Field Values

---


### FLUX\_INTERVAL\_FILE

```
public static final java.lang.String FLUX_INTERVAL_FILE
```

:   The keyword used to denote a flux interval input file.

    **See Also:**: Constant Field Values


**Constructor Detail**

### IntervalVariationManager

```
public IntervalVariationManager()
```


**Method Detail**

### getCvInputFile

```
public static FluxIntervalInputFile getCvInputFile(ManagerInput<java.lang.String,java.lang.String> input,
                                                   java.io.PrintWriter out,
                                                   Fluxome fluxome)
```

:   Returns the constraint variation input with the name denoted by
    `CV_FILE` in the provided manager input.

    :   **Parameters:**: `input` - the input containing a value for `CV_FILE`.: `out` - the print writer to which a status message will be printed.: `fluxome` - the fluxome to which the constraints correspond. **Returns:**: the constraint variation input with the name denoted by `CV_FILE` in the provided manager input.

---


### getCvInputFile

```
public static FluxIntervalInputFile getCvInputFile(java.lang.String fileName,
                                                   Fluxome fluxome)
```

:   Returns the constraint variation input file with the provided name.

    :   **Parameters:**: `fileName` - the name of the constraint variation file to open.: `fluxome` - the fluxome to which the constraints correspond. **Returns:**: the constraint variation input file with the provided name.

---


### getSvInputFile

```
public static FluxIntervalInputFile getSvInputFile(ManagerInput<java.lang.String,java.lang.String> input,
                                                   java.io.PrintWriter out,
                                                   Fluxome fluxome)
```

:   Returns the space variation input with the name denoted by
    `FLUX_INTERVAL_FILE` in the provided manager input.

    :   **Parameters:**: `input` - the input containing a value for `FLUX_INTERVAL_FILE`.: `out` - the print writer to which a status message will be printed.: `fluxome` - the fluxome to which the constraints correspond. **Returns:**: the space variation input with the name denoted by `FLUX_INTERVAL_FILE` in the provided manager input.

---


### getIntervalInputFile

```
public static FluxIntervalInputFile getIntervalInputFile(java.lang.String fileName,
                                                         Fluxome fluxome,
                                                         MapFormat<LinearComb<java.lang.String>,Interval> mapFormat)
```

:   Returns the flux interval input file with the provided name.

    :   **Parameters:**: `fileName` - the name of the flux interval file to open.: `fluxome` - the fluxome to which the constraints correspond.: `mapFormat` - the parser. **Returns:**: the flux interval input file with the provided name.

---


### getIntervalInputFile

```
public static FluxIntervalInputFile getIntervalInputFile(java.lang.String fileName,
                                                         Fluxome fluxome)
```

:   Returns the flux interval input file with the provided name.

    :   **Parameters:**: `fileName` - the name of the flux interval file to open.: `fluxome` - the fluxome to which the constraints correspond. **Returns:**: the flux interval input file with the provided name.


---


|  |  |  |
| --- | --- | --- |
| **Overview** \| **Package** \| **Class** \| **Use** \| **Tree** \| **Deprecated** \| **Index** \| **Help** | |  |
| **PREV CLASS**   **NEXT CLASS** | **FRAMES**    **NO FRAMES**     **All Classes** |  |
| SUMMARY: NESTED \| FIELD \| CONSTR \| METHOD | DETAIL: FIELD \| CONSTR \| METHOD |  |


---
